# Big data analytics and smart cities: applications, challenges, and opportunities

**DOI:** 10.3389/fdata.2023.1149402

**Published:** 2023-05-12

**Authors:** Eugenio Cesario

**Affiliations:** University of Calabria, Rende, Italy

**Keywords:** smart cities, big data analysis, crime forecasting, mobility patterns, trajectory mining, COVID-19

## Abstract

Urban environments continuously generate larger and larger volumes of data, whose analysis can provide descriptive and predictive models as valuable support to inspire and develop data-driven Smart City applications. To this aim, Big data analysis and machine learning algorithms can play a fundamental role to bring improvements in city policies and urban issues. This paper introduces how Big Data analysis can be exploited to design and develop data-driven smart city services, and provides an overview on the most important Smart City applications, grouped in several categories. Then, it presents three real-case studies showing how data analysis methodologies can provide innovative solutions to deal with smart city issues. The first one is an approach for spatio-temporal crime forecasting (tested on Chicago crime data), the second one is methodology to discover mobility hotsposts and trajectory patterns from GPS data (tested on Beijing taxi traces), the third one is an approach to discover predictive epidemic patterns from mobility and infection data (tested on real COVID-19 data). The presented real-world cases prove that data analytics models can effectively support city managers in tackling smart city challenges and improving urban applications.

## 1. Introduction

In several reports the twenty-first century is frequently referenced as the “Century of the City” (Nat, 2010; Zheng et al., 2014). The main reason of this definition is due to the unprecedented global migration of people into urban areas that is happening nowadays (Cesario et al., 2016b). In fact, the world is currently experiencing the largest urban growth seen in history so far, and it is rapidly urbanizing. For example, several United Nations reports state that urban population is expected to grow to 4.98 billion in 2030 (UNR, 2014). As a matter of fact, this means that around sixty percent of the global population will be living in cities by 2030.

The above described urbanization process is transforming the organization of cities, making urban environment bigger and more crowded, and it is causing significant environmental, economic and social transformations. In fact, on the one hand it is bringing modernization in people's lives, and providing challenging opportunities offered in urban areas; on the other hand, it is bringing new issues in city management, such as increasing traffic congestion, large-scale resource planning, air pollution, crime rising, energy consumption, water quality, etc. (Zheng et al., 2014; Altomare et al., 2019; Cesario, 2019; Piaggesi et al., 2022).

Considering the complex and dynamic settings of cities, just a few years ago it seemed nearly impossible tackling the aforementioned challenges. However, the pervasive presence of sensors in cities, as well as the availability of large-scale computing infrastructures (Cesario et al., 2013; Al Nuaimi et al., 2015), has been facilitating the gathering of huge volume of data (i.e., electricity/water consumption, air quality, mobility, etc.) (Cesario and Talia, 2008; Bejan et al., 2010; Herrera et al., 2010). Such big collections of urban data, containing rich knowledge about a city, represent a valuable opportunity to achieve improvements in management issues and urban policies.

Recently, several research activities have been focused to the development of Smart City services and applications, with the aim of making our cities more and more livable and efficient (Potgieter et al., 2021; Yan et al., 2021; Cesario et al., 2022). In particular, a Smart City is defined as “an urban environment where public issues are addressed via ICT-based solutions on the basis of municipality and multi-stakeholder based partnership” (EUP, 2017). Also, modern technological infrastructures and computer systems can allow the implementation of efficient facilities and smart services, thus improving the quality of citizens' lives and naturally enabling the transition to smarter and smarter cities. To this purpose, data analytics and machine learning can provide an important contribution to the development of smart cities. In fact, such disciplines can offer useful algorithms and tools for gathering, aggregating, associating and classifying data; such tools can support the analysis of urban data and support the extraction of useful knowledge for citizens and decision makers. Considering such an abundance of data, the acquisition and analysis of urban data is crucial to discover descriptive and predictive data-driven models, which can support city managers in tackling the major issues that cities face, including, e.g., air pollution, virus diffusion, human mobility, traffic flows, crime forecasts, etc. This has enabled the development of innovative solutions and new smart city applications, exploiting urban data analysis techniques and methodologies, have been implemented world-wide (Zheng et al., 2014; Al Nuaimi et al., 2015; Potgieter et al., 2021; Yan et al., 2021; Cesario et al., 2022).

This paper introduces how Big Data analysis can be exploited to design and develop data-driven smart city services. Then, it provides an overview on the most important Smart City applications, grouped in several categories. Also, a detailed and critical comparison among the approaches proposed in the literature, in terms of applications and adopted methodologies, is sketched in a table. Finally, it presents three real-case studies we recently worked on, showing how data analysis methodologies can provide innovative solutions to deal with smart city issues. The first one is a methodology based on spatial analysis and auto-regressive models for spatio-temporal crime forecasting, which has been tested on crime events occurred in Chicago (Catlett et al., 2019). The second one is methodology to discover mobility hotsposts and trajectory patterns from GPS data, which has been tested on Beijing taxi traces (Cesario et al., 2017). The third one is an approach to discover spatio-temporal predictive epidemic patterns from mobility and infection data, whose experimental evaluation has been carried out on real-world COVID-19 data. (Canino et al., 2022a). The presented real-world cases are aimed to show three example where data analytics models can provide effectively valuable support for city managers in tackling smart city challenges, to improve urban applications and citizens' lives.

The paper is structured as follows. Section 2 provides an overview of the most important Smart City applications. Section 3 describes an approach for crime predictions. Section 4 shows a method to discovery mobility hotspots and frequent mobility patterns. Section 5 describes an approach to discover predictive epidemic models from infections and mobility data. Finally, Section 6 concludes the paper, summarizes its contribution and depicts further research challenges.

## 2. State of art of smart city applications

In the last years an increasing number of innovative services and applications, exploiting urban data analysis, have been implemented in our urban environment to build smart cities. We report here a brief descriptions of the most important applications, grouped in several categories.

### 2.1. Smart transportation

Several applications, aimed at improving city mobility (i.e., taxi services, bike sharing, smart parking, human mobility, etc.) have been proposed in literature (Bejan et al., 2010; Herrera et al., 2010; Cesario, 2019). To develop these tasks, commuting and traffic data are a fundamental source of data, whose joint analysis can discover descriptive and predictive mobility models. Mobility data can also be gathered through a network of sensors distributed in the city, GPS on-vehicle devices, smart traffic lights, etc., whose pervasive presence in modern cities is becoming more and more popular. In urban environments, private and public mobility systems can benefit from mobility knowledge models, which can be used in particular to anticipate or resolve traffic problems. For example, (Bejan et al., 2010) describe a research study aimed at discovering historical traffic patterns to suggest fast driving routes at real-time, while Herrera et al. (2010) and Castro-Neto et al. (2009) propose specific algorithms to predict real-time traffic flows and forecast future traffic conditions on individual road segments. Also, some solutions for improving the efficiency and reliability of public transportation systems are proposed in Al Nuaimi et al. (2015); Zheng et al. (2014); in particular, such papers describe how Big Data analysis can be profitable exploited to perform real-time arrival time forecasting of buses, and to predict bicycle flows for bike sharing system operators. Finally, several solutions have been also proposed to improve taxi services. For example, Yuan et al. (2013) describe a system (for taxi drivers) that suggests the most likely routes (and locations) to pick up the next passengers quickly, while Ma et al. (2013) report the description of a system that maximizes the profit of ride-sharing trips by appropriately choosing the pick-up passengers on the basis of capacity, time, and money constraints. Recently, in Li et al. (2019) has been described the design of a large-scale urban vehicular network framework for IoT in Smart Cities, aimed at providing more reliable and predictable wireless connections in metropolitan areas. Liao et al. (2019) proposed a vehicle mobility-based geographical migration model, for an efficient management of vehicular computing resources in fog computing-enabled smart cities. Pan et al. (2019) exploit DE-BP (differential evolution back propagation) neural network models to predict mobile telecommunication traffic in a smart city, to increase upstream and downstream bandwidth, and improve reliability and quality of wireless-connected city services. A distributed system for collaborative gathering of traffic data is proposed in Fujihara (2020), where special beacon devices are deployed along road segments to collect traffic data; such a distributed beacon system is exploited for real-time detection of anomalies, such as traffic jam and accidents. Brisimi et al. (2016) propose a machine learning approach, based on data collected trough smart phones, to classify roadway obstacles into predefined categories and support quick decisions to solve anomalies.

### 2.2. Smart healthcare

Several solutions to be adopted in the healthcare domain, leveraging on data analysis to improve hospitalized patient's lives, have been proposed in Al Nuaimi et al. (2015); Zheng et al. (2014). For example, real-time monitoring systems can collect real-time data (sleeping patterns, cholesterol, blood pressure) through smart devices, and they directly communicate with hospital ICT systems to integrate a comprehensive patient history and to allow timely responses to possible health issues. Muhammed et al. (2018) describe a framework for preventive, and personalized healthcare services, leveraging edge computing, deep learning, big data, high-performance computing (HPC), and the Internet of Things (IoT). In Samani and Zhu (2016), an ambulance robot has been designed and developed, which brings along an automated external defibrillator (AED) to facilitate manual and/or autonomous functioning, to promptly deal with cardiac arrest events and save people lives in smart cities.

### 2.3. Smart energy

Data analysis is also profitable exploited to deal with energy consumption issues, which are becoming more and more important due to the rapid urbanization phenomenon. In fact, big cities are demanding for increasing requests of energy, and scientists and engineers are continuously working to design technological solutions for energy-efficient infrastructures, with the aim of decreasing city-scale energy costs and reducing energy consumptions (Zheng et al., 2014; Ullah et al., 2017; Altomare et al., 2019). For example, Zheng et al. (2014) describe how predictive models can forecast high-demand or low-demand energy periods, or time windows allowing an high availability of renewable power. Such knowledge can support a more efficient and effective usage of energy in urban and sub-urban environments, also when there are some constraints related to community-assigned energy usage limits. Ullah et al. (2017) describe an energy (and congestion)-aware routing metric for smart meter networks to be deployed in smart cities. In particular, advanced metering infrastructures (AMIs) can exploit this metric to minimize power consumption and efficiently use the residual energy and queue utilization of neighboring nodes. Altomare et al. (2019) describe an energy-aware solution, driven by predictive data mining models, for energy-efficient allocation of virtual machines in Cloud systems. In particular, migrations are driven by the forecast of the future computational needs of each virtual machine, in order to efficiently allocate those on the available servers, thus achieving good benefits in terms of energy saving.

### 2.4. Smart environment

The collection and analysis of environmental data are very important to understand how natural phenomena (i.e., global warming, drought, torrential rains) are influenced by other factors, such as urban air quality, pollution, land uses, etc. Moreover, a more efficient management of energy utilization can improve agriculture effectiveness and crops efficiency (Al Nuaimi et al., 2015). Recently, Liv (2017) present a research study describing the design and development of a real-time control system based on weather and transportation data, aimed at forecasting how weather conditions influence taxy demand. Also, in Zheng et al. (2014) authors study how people's physical and mental health issues are affected by noise and pollution densities.

### 2.5. Smart safety and security

Data analytics can be successfully applied on data related to crimes, pandemics, terrorism attacks, to provide insights and knowledge about threats to public order and security. In fact, police departments are collecting and storing criminal events in databases, each one described by several features (time, location, type, etc.). The analysis of such crime data can enable the extraction of crime knowledge models, which can be exploited to forecast the number of criminal events that will happen in specific areas of the city (Zheng et al., 2014). For example, the papers Cesario et al. (2016a) and Catlett et al. (2019) describe a methodology (and its application on real-world data) aimed at understanding crime patterns and trends, to detect crime knowledge models that can detect the crime hotspots and the number of crimes will happen in each specific hotspot. These models can be profitably exploited to anticipate criminal activity, and to optimize the distribution of police officers over the territory, to improve patrol routes, etc. Some projects and computing architectures for the prediction of natural disasters are described in Cesario and Talia (2010, 2012). In particular, some frameworks are specifically aimed at ground shaking forecasting and earthquakes predictions. Despite such approaches do not achieve good performance yet, an important research effort is invested on these topics, whose results can give an opportunity to save lives and resources. Jamshidi et al. (2020) describe a technique to detect malicious nodes (performing node replication attacks) in mobile Wireless Sensor Networks deployed in smart cities. In particular, watchdog nodes collaborate to measure sensor nodes' speed in the environment, marking nodes moving faster than usual (in different regions of the network) as malicious, thus making an attempt on the network security. Ali et al. (2020) deal with security threats related to the Internet of Drones (IoD), whose applications are steadily increasing in many military and civilian-based scenarios. In particular, authors propose a technique to improve the communication security of sensitive data collected through drones, especially the surveillance data in smart cities using the current cellular networks.

Tables 1, 2 report a more detailed and critical comparison among several approaches proposed in the literature. The comparison takes into account several features, as detailed in the following:

- *Domain*. This feature differentiates the approaches on the basis of the domain they are applied on. In particular, Table 1 presents a summary of techniques aimed at smart transportation, while Table 2 shows a summary related to smart healthcare, smart energy and smart safety/security.
- *Application use case*. This feature differentiates the approaches on the basis of the use cases they have been tested on. As it is shown in the two tables, the applicative scenarios are very heterogenous, ranging from traffic pattern detection to road anomalies forecasting, from personalized healthcare services to automatic defibrillator robot, from crime forecasting to securing Internet of Drones communications.
- *Methodologies and techniques*. This feature differentiates the algorithms on the basis of the methodology and/or techniques used to address the faced task. Some approaches exploit classification, regression and clustering models, while others are based on deep learning and statistical learning techniques. There are also some recent approaches, based on blockchain and Internet of Drones technologies.

**Table 1:** Comparison of several approaches proposed in literature for smart transportation.

**References**	**Domain**	**Application use case**	**Approaches–techniques**
Bejan et al. (2010)	Transportation	Discovering historical traffic patterns to suggest fast driving routes at real-time	Quantile regression, Spline function
Herrera et al. (2010)	Transportation	Predicting real-time traffic flows to improve urban mobility	Sampling strategy, statistical learning
Castro-Neto et al. (2009)	Transportation	Forecasting future traffic conditions on individual road segments	Support vector machines for Regression, Holt exponential smoothing
Zheng et al. (2014)	Transportation	Predicting bicycle flows to improve bike sharing systems	Knowledge fusion across heterogeneous data, urban data visualization
Yuan et al. (2013)	Transportation	Recommendation system for taxi drivers, to suggest the most likely next passenger's pickup-up location	Density-based clustering, ensemble classification
Li et al. (2019)	Transportation	Large-scale urban vehicular network framework for IoT in Smart Cities, to improve wireless connectivity	Statistical analysis, location-based urban vehicle network
Ma et al. (2013)	Transportation	Decision support system to predict ride-sharing trips, aimed at appropriately choosing the pick-up passengers on the basis of capacity, time, and money constraints.	Scheduling algorithm, spatio-temporal index data structure, shortest path calculation strategies
Liao et al. (2019)	Transportation	A vehicle mobility-based geographical migration model for an efficient management of vehicular computing resources in fog computing-enabled smart cities.	IoT computing, fog-enabled geographical migration scheme for computing resources, simulated annealing, Dijkstra algorithm
Pan et al. (2019)	Transportation	Predicting mobile telecommunication traffic, to improve reliability and quality of wireless-connected city services	Differential evolution back propagation (DE-BP) neural network
Fujihara (2020)	Transportation	A distributed system for collaborative management of traffic data, for real-time detection of traffic jam and accidents.	Blockchain technology, distributed consensus algorithms, geographical proximity analysis
Brisimi et al. (2016)	Transportation	Classification of roadway obstacles into predefined categories, to support quick decisions and solve road anomalies.	Classification, clustering

**Table 2:** Comparison of several approaches proposed in literature for smart healthcare, smart energy, and smart safety and security.

**References**	**Domain**	**Application use case**	**Approaches–techniques**
Al Nuaimi et al. (2015)	Healthcare	Prompt responses to possible health issues through real-time data monitoring and analysis, performed by smart devices directly communicating with hospitals	Smart network infrastructure and big data anlaysis
Samani and Zhu (2016)	Healthcare	Robotic outer defibrillator vehicle to promptly deal with cardiac arrest events	Ambulance robot, robotic systems, vehicle-to-vehicle communication
Muhammed et al. (2018)	Healthcare	Performing preventive, and personalized healthcare services	Internet of things and deep learning techniques
Altomare et al. (2019)	Energy	Energy-efficient allocation of virtual machines in Cloud systems, driven by predictive data mining models	Classification, regression
Ullah et al. (2017)	Energy	Energy (and congestion)-aware routing metric to minimize power consumption and to efficiently use the residual energy in smart cities	Nearest neighbors, RPL routing techniques
Catlett et al. (2019)	Safety	Spatio-temporal prediction of crime patterns and trends, to detect crime hotspots and location-based regressive models	Density-based clustering, Regression
Jamshidi et al. (2020)	Safety	Detecting malicious nodes in mobile wireless sensor networks	Algorithms based on watchdog nodes to improve network security issues
Ali et al. (2020)	Safety	Securing sensitive data collected for smart cities surveillance through Internet of Drones	A scheme exploiting lightweight symmetric key natives and symmetric encryption/decryption operations

## 3. An approach to perform spatio-temporal crime predictions in smart cities

As described in Section 2, several research studies have been devoted to propose solutions aimed at improving the security in our cities by exploiting data analytics. Among the approaches proposed in literature, we focus here on the study presented by Catlett et al. (2019), aimed at extracting crime predictors to perform spatial and temporal forecasting of criminal events. The approach is based on spatial analysis and auto-regressive models, with the aim to first automatically detect crime hotspots (i.e., high-risk crime regions) in urban areas and to perform a reliable forecast of crime trends in each hotspot. As described in the following, the algorithm builds a set of spatio-temporal crime forecasting models, i.e., a set of crime hotspots with associated predictive models estimating the number of crimes likely to occur in its associated hotspot. The accuracy and effectiveness of the approach have been tested on two real-world scenarios, i.e., crimes occurred in Chicago and New York City (we will show here the results on the first use case).

### 3.1. Approach

**Figure 1:**
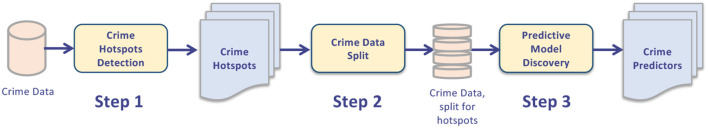
Spatio-temporal crime prediction steps.

Let *T* = < *t*_1_, *t*_2_, …, *t*_*H*_ > be an ordered timestamp list. Let D be *D* = {*D*_1_, *D*_2_, …, *D*_*N*_} a dataset collecting crime records, where each *D*_*i*_ is a crime instance described by its xy-position (coordinates of the place the crime occurs) and timestamp (time the crime happens at). The goal of the approach is twofold. First, extract a set of *crime hotspots* (or crime dense regions), where a *crime hotspot* is a spatial area which criminal events occur in with an higher density than other areas in the city. Second, extract a function that can forecast the number of crimes in each detected *crime hotspot*. The general idea and the main steps of the approach are sketched in Figure 1, through a graphic representation of the whole process. The algorithm takes in input a dataset of crimes occurred in an urban area, and returns in output a knowledge model composed by a set of crime-dense regions with associated crime predictors. The workflow is composed of three main steps, as described below.

**Step 1: Crime hotspots detection**. Initially the algorithm performs a spatial analysis over the input data, with the aim to detect *crime dense regions*, i.e., areas (i.e., polygons, blobs) whose density of crime events is higher than adjacent areas. The goal of this step is to reduce the spatial granularity of the analysis, in order to conduct the further steps considering the detected dense regions, and not the single points occurring in the dataset. The task is modeled as a geo-spatial clustering process, by running a density-based clustering algorithm. A good property of this approach is that it automatically traces the boundaries of the detected clusters, without relying on any pre-fixed division in areas. At the end of this step, the algorithm returns *K* clusters where *K*, depending on the specific adopted clustering algorithm, can be automatically detected or fixed a-priori. In this scenario each cluster represents a detected crime dense region.

**Step 2: Crime data splitting**. After the detection of the crime hotspots, the next step is a data transformation task, consisting in a *spatial data splitting* of the original crime data. More specifically, the set of all events occurring in locations belonging to the *i*^*th*^ crime region are transformed (considering their timestamp) in a time series and gathered in the *i*^*th*^ output dataset, for *i* = 1, …, *K*. The final result of this step is a set of *K* different time series data sets, where each one is the time series of all events occurred in its associated area.

**Step 3: Crime predictive models discovery**. This final step has the goal to extract, for each crime dense region *CDR*_*i*_, a specific crime prediction model for *CDR*_*i*_. In particular, predictive models are trained on the time series crime data built during the previous step, to detect predictive regressive models aimed at forecasting the number of crime events that will happen in each specific area. As regression model, ARIMA models (i.e., a composed technique based on auto-regression, moving average and difference modeling) (Catlett et al., 2019) have been exploited. As a result of such a task, several crime predictors (one for each crime hotspot) are obtained.

### 3.2. Experimental results

As case study to test the effectiveness and performance of the approach described above, in Catlett et al. (2019) is presented as case study the analysis of crimes within a large area of Chicago, involving about two million crime events over a period of 16 years. As aforementioned, the application of the approach in a real case scenario has two main objectives: (*i*) discovering the most significant crime dense regions, and (*ii*) extracting effective predictive models. The integration of these two models can be exploited to estimate the areas where future criminal events are likely to happen, and the estimated number of crimes to occur.

**Figure 2:**
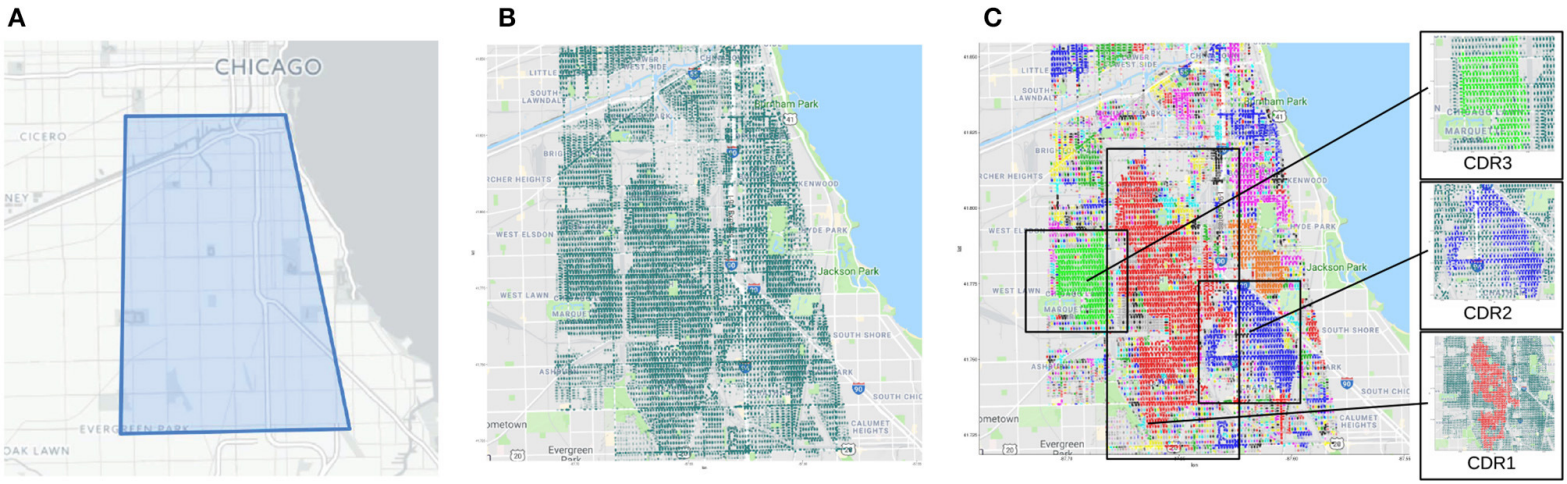
Selected area of Chicago, geolocalized crime events (2001–2016) and crime dense regions. **(A)** Polygon of the geographic area. **(B)** Geo-localized crime events. **(C)** Detected crime dense regions.

The geographic area of Chicago the tests have been performed on is shown in Figure 2A, while the geo-localized crime events are reported in Figure 2B. The selected area has a perimeter of about 52*KM* and its area is approximately 135*KM*^2^. For the tests, all crime events occurred within the bounded area and happened from January 2001 - December 2016 have been collected, whose total number was around 2 million instances. Crime dense regions have been detected by applying a DBSCAN-based algorithm. in particular, as described in Catlett et al. (2019), the algorithm assigns a higher weight to recent crime events by exploiting a decay factor integrated in the distance computation. Figure 2C illustrates such regions, with each one represented by a distinct color. It is interesting to note that this image shows how crime incidents are grouped according to a density criterion; for instance, the algorithm identifies eight significant crime regions that are easily distinguishable by different colors: a large crime region (in red) located in the area's center, along with seven smaller areas (in green, blue, and light-blue) on the left and right side, all corresponding to zones characterized by an high density of crimes.

On the left side of Figure 2C, the three areas with the highest crime rates (*CDR1, CDR2*, and *CDR3*) are zoomed-in. Throughout the entire territory, there are numerous additional smaller areas that reflect relatively localized high-crime zones. The algorithm's further steps are the geographic data splitting of the initial crime data set (to create a time series for each identified dense zone) and the training of local crime predictors for each identified dense region, which have been trained by exploiting ARIMA models. An aspect to be considered here is that, the auto-regressive models of the three largest crime dense regions are characterized by different parameters. This means that each area presents specific crime trends and patterns.

**Figure 3:**
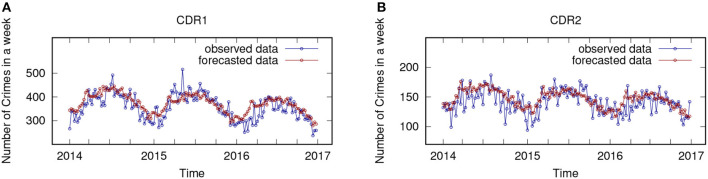
Number of crimes observed and forecasted (blue and red lines) on two crime dense regions. **(A)** Crime Dense Region 1 (CDR1). **(B)** Crime Dense Region 2 (CDR2).

The experimental evaluation of the regressive functions performance has been assessed on the test set (crimes occurred from 2014 to 2016) in the available dataset. In particular, the analysis has been performed considering the crime dense regions, and for comparison purposes, also the whole area. Thus, ARIMA models have been extracted for each crime dense region and for the whole area, in order to test their forecasting accuracy to predict the number of crimes that are likely to happen in each region and in the whole area, week by week. Figure 3 shows observed and forecasted data (plotted in blue and red, respectively) for the test set period, plotting the curves for the two largest crime dense regions (CDR1 and CDR2) detected during the analysis. Considering the whole test set period, we notice that the forecasted curve (red) adheres very well to the observed curve (blue). Finally, the paper Catlett et al. (2019) reports the values of several error measures, for the whole area and the three largest crime dense regions detected. The results are reported by considering three different horizons, i.e., 1, 2, and 3 years; in particular, the average MAPE (Mean Absolute Percentage Error) forecasting error is 9.62% for the first year, 11.90% for the second year, and 18.66% for the third year. These values show overall good prediction accuracy and very interesting predictive performance. A comparative analysis between the forecasting performance of ARIMA models vs. three state-of-the-art regression algorithms [i.e., RandomForest (Breiman, 2001), REPTree (Witten Ian, 2011), ZeroR (Nasa and Suman, 2012)] is reported in Table 3. In particular, the table summarizes the results of the comparison, showing the achieved MAE (Mean Absolute Error) and MAPE, vs. different prediction horizons (1-, 2-, and 3-year forecasts). By observing the values in the table, we can conclude that the ARIMA approach generally achieves greater accuracy than other algorithms. Also, shorter the time horizon, higher the forecasting accuracy. These results confirm the appropriateness of the autoregressive model and its good performance in the crime prediction domain. More detailed results can be found in Catlett et al. (2019).

**Table 3:** Comparative analysis among several approaches, evaluating the Mean Absolute Error (MAE) and Mean Absolute Percentage Error (MAPE) of the crime dense regions, vs. several time horizons.

	**MAE**				**MAPE**			

**Time**	**ARIMA**	**Random Forest**	**RepTree**	**ZeroR**	**ARIMA**	**Random Forest**	**RepTree**	**ZeroR**
2014	30.51	44.20	57.47	97.15	9.62	16.68	22.86	39.90
2015	39.54	57.24	64.54	109.24	11.90	18.75	29.49	45.66
2016	46.47	68.04	71.86	117.83	18.66	21.47	34.16	53.66

## 4. Discovery of mobility patterns from urban mobility data

An approach for mobility data analysis, named *TPM (Trajectory Pattern Miner)*, aimed at the discovery of trajectory patterns from GPS data, is proposed in Cesario et al. (2017). The inspiring idea and motivations of the work is that the detection of mobility (or trajectory) patterns is a basic knowledge to be exploited for the implementation of more complex tasks. A first example is represented by *next location prediction*, that is the prediction of the possible future location of a moving object, whose information can be used to pre-fetch or anticipate the delivery of some service in that location. Another case is the *intelligent traffic management*, that is, predicting traffic congestion patterns, which can be exploited to adopt improvements to the urban transportation model and reduce the vehicular traffic. A third example is represented by *travel recommendations*, that is, predicting the top interesting locations and travel sequences among locations, and exploit such information to recommend the best routes and itineraries that tourists can follow to visit a given location. The approach has been evaluated on a real-world case study, i.e. a dataset composed of GPS points tracing the mobility of taxis in the urban area of Beijing.

### 4.1. Approach

Let be *T* = < *t*_1_, *t*_2_, …, *t*_*H*_ > an ordered list of timestamps. A *trajectory dataset* *TD* = {τ_1_, τ_2_, ..., τ_*H*_} is a set of trajectories, where each trajectory τ_*h*_ = < (*x*_1*h*_, *y*_1*h*_, *t*_1_), …, (*x*_*nh*_, *y*_*nh*_, *t*_*h*_) > is a list of *n* triples reporting the xy-position and timestamp. The goal of the approach is twofold. First, discover a set of *dense regions*, where a dense region is an area of points that is more frequently visited by object's trajectories with respect to other areas. In particular, $R_{t}^{j}$ represents the *j*^*th*^ dense region at the time *t*. Second, discover a set of *trajectory patterns*, where each trajectory pattern *tp* is in the form: $tp:R_{t_{1}}^{j_{1}},R_{t_{2}}^{j_{2}},\ldots ,R_{t_{r}}^{j_{r}}\overset{}{\underset{}{\to }}R_{t_{s}}^{j_{s}}$, representing frequent sequences occurring in the dataset among the involved dense regions.

**Figure 4:**
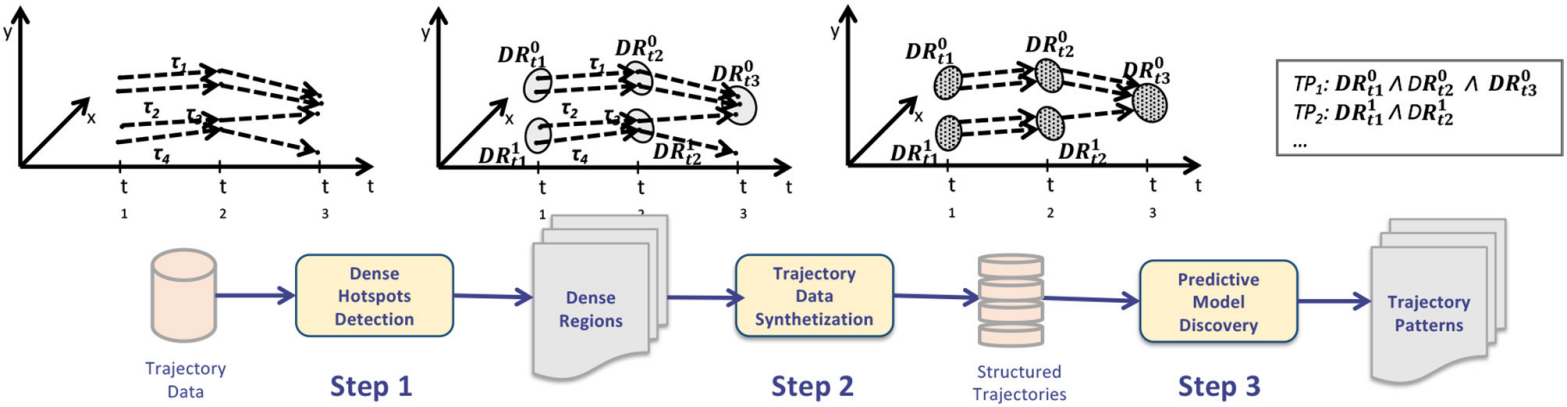
Trajectory patterns detection steps.

The workflow and main steps of the approach, designed to discover mobility patterns from GPS data, is depicted in Figure 4. The algorithm receives in input trajectory data traced by objects (cars, buses, humans, etc.) in a city, and returns a set of (*i*)mobility hotspots (areas more densely passed through ones) and (*ii*)mobility patterns. The method consists of (*i*)discovering urban dense regions of interest (more densely passed through ones) and (*ii*) discovering mobility patterns among those regions. Figure 4 sketches the general idea of the algorithm through a graphic representation of the whole process as a sequence of three main steps, as described below.

**Step 1: Frequent Regions Detection**. Initially the algorithm detects a set of *mobility dense regions* from the original dataset, i.e., a set of raw mobility routes traced by drivers during their daily activities. The goal is to detect, for each timestamp, the spatial areas (or regions) that are more densely passed through than others. This task has been performed by geo-spatial clustering algorithm. In particular, *H* clustering instances are executed, each one taking in input points visited at the *h*^*th*^ timestamp. At the end of this step, *H* clustering models are returned, whereas the *t*_*h*_-model corresponds to the dense regions detected at the *t*_*h*_-timestamp. In figure, the *j*^*th*^ dense region at time *t*_*h*_ is represented by $DR_{t_{h}}^{j}$ .

**Step 2: Trajectory Data Synthetization**. This step aims at building a structured trajectory dataset; more precisely, this step converts the raw data (movements between points) into movements between dense regions (structured data). This is done by processing the original dataset and substituting each trajectory by the dense region it belongs to (such information is modeled in the dense region model set). The transformation consists in replacing each point of the original dataset by the region it belongs to.

**Step 3: Trajectory Patterns Extraction**. By evaluating the trajectories of dense regions detected in the previous phase, this step aims to extract trajectory patterns, in the form of sequential patterns. The dense regions trajectory data is specifically subjected to a trajectory pattern extraction technique in order to extract trajectory patterns from it. The ultimate mining model is composed of a collection of associative rules describing the spatio-temporal relationships between the movement of the users under examination.

### 4.2. Experimental results

The experimental evaluation of TPM has been performed on T-Drive (Yuan et al., 2011), a real-world dataset collecting the GPS-detected trajectories of taxies driving in the city of Beijing. Specifically, T-Drive contains 10,357 instances (i.e., taxi trajectories), cumulatively covering a distance of almost 9 million kilometers. The total number of GPS points collected in the data amount to about 15 million records.

Given the mobility input dataset, it was necessary to perform a pre-processing task to clean, select and transform instance data, to make it suitable for the further analysis. First, a cleaning step has been done on the collected data to remove all the points with unreliable or evident wrong positions (due to gathering issues). Then, geo-localization errors, i.e. points outside this area, have been handled by selecting only data points falling in a bounded area limiting the city. Finally, the data has been transformed by partitioning each trajectory in a daily route, to deal with daily patterns inside data. After the execution of such pre-processing steps, the final dataset results a collection of about more than 61,000 daily trajectories, where each one contains the set of geo-localized points traced by a single taxi during a day. The size of the final dataset is about 882 MB. The results of the analysis carried out on such a dataset are reported in the following, by showing the detected (*i*)*dense regions* (representing mobility hotspots or the most congested areas of the city) and (*ii*)*mobility patterns* with respect to different timestamps.

#### 4.2.1. Discovered dense regions

**Figure 5:**
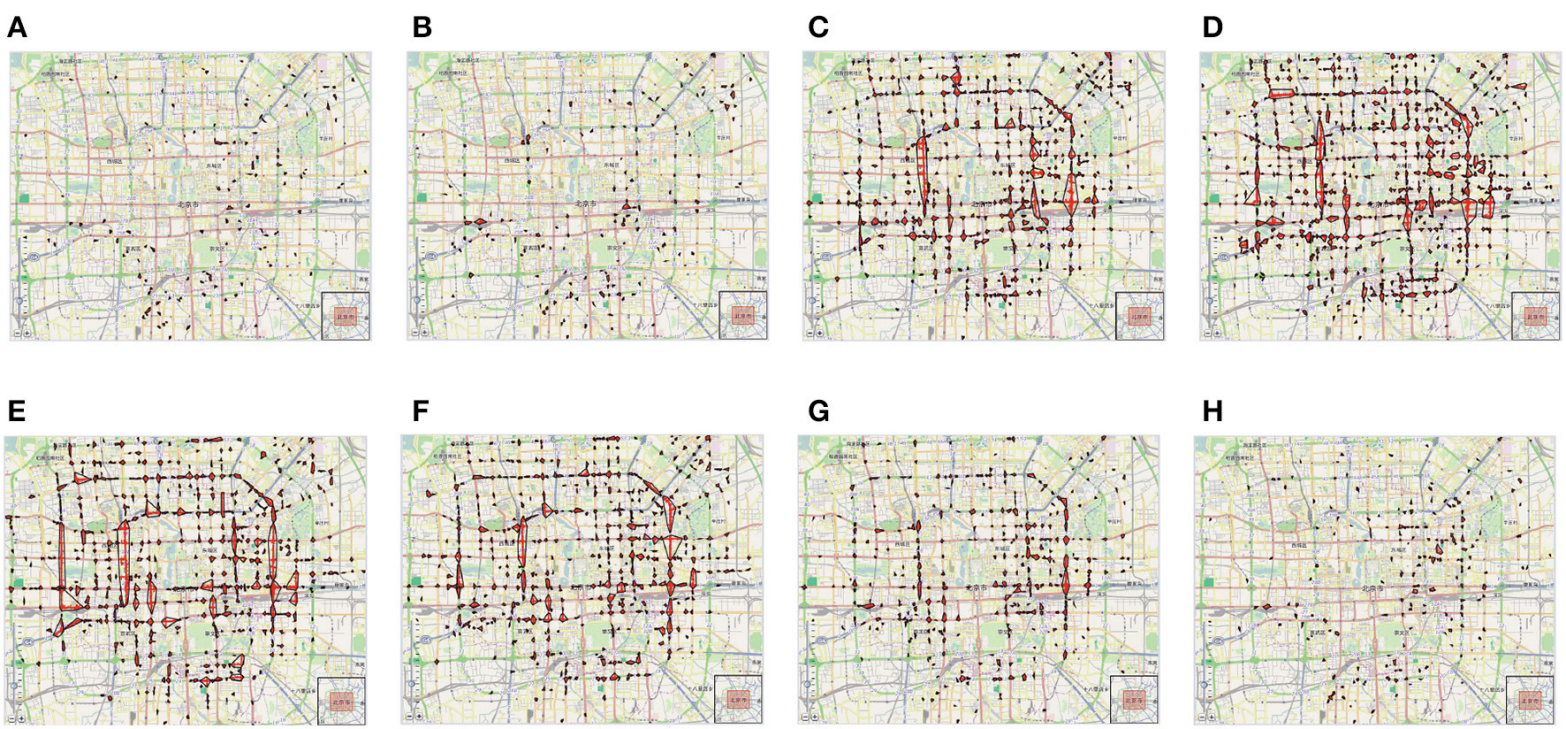
Dense regions discovered in T-Drive, w.r.t. several time windows of the day. **(A)** 6:00 a.m. **(B)** 9:00 a.m. **(C)** 12:00 p.m. **(D)** 3:00 p.m. **(E)** 6:00 p.m. **(F)** 9:00 p.m. **(G)** 12:00 a.m. **(H)** 3:00 a.m.

Figure 5 shows the dense regions discovered in T-Drive, for different 3-h time windows of the day. By observing the figures, we can observe that the traffic congestion and the taxi mobility change over the day. For example, during the early morning (from 6 to 9 a.m.), a few dense regions localized in West and South areas of the cityt are detected (Figures 5A, B). Then, from the late morning to the evening, the traffic increases in several areas and the distribution of vehicles increases its variability. In particular, we can observe (Figures 5C–F) that, from 12:00 PM to 9:00 PM, the concentration of taxies is high in many regions of the city. We can recognize, from the image, the main roads and highways that are used during these times: an highway toward the airport, a circular highway around the city center and several highways crossing the central area of the city. Finally, the density of driving taxies strongly decreases during the night (Figures 5C, D); however, there are some parts of the city where the density of cars is still high.

#### 4.2.2. Discovered mobility patterns

**Figure 6:**
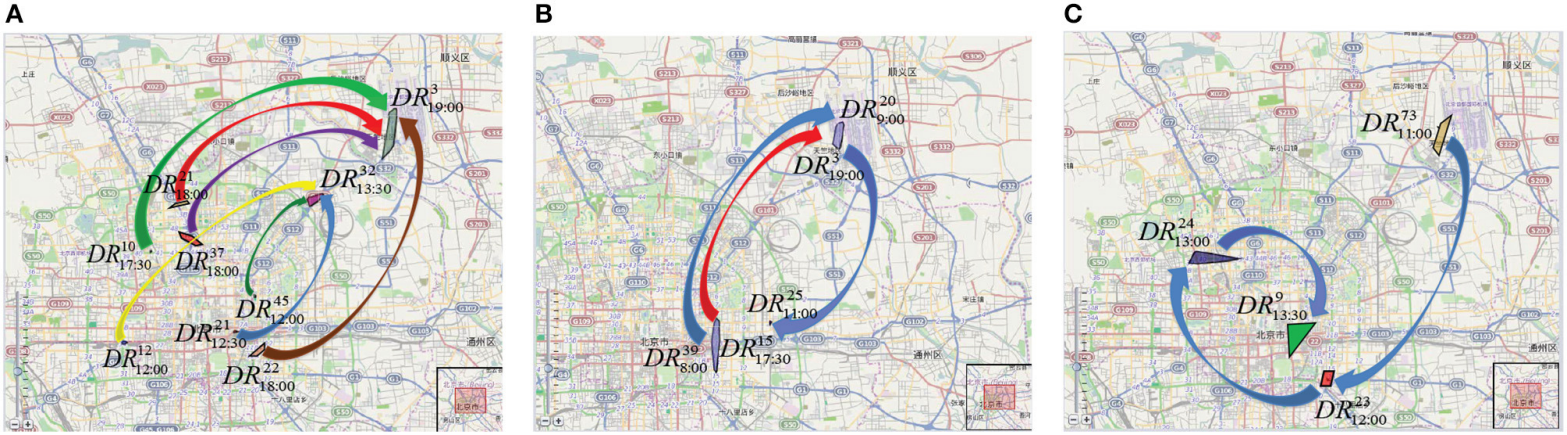
Travel patterns discovered in T-Drive by the TPM algorithm. **(A)** From the city center to the airport. **(B)** From the city center to sub-urban east area of the city. **(C)** From the airport to the train station.

Examples of the most frequent mobility patterns found in T-Drive by the TPM algorithm are displayed in Figure 6. We focus our attention on routes surrounding the city center and those leading from the city center to important locations like the airport and train stations, in order to identify the most popular itineraries. Figure 6A illustrates the primary routes taxi drivers take to head out of the city and toward the airport. It is clear that although the starting points for the taxies in the city center are widely dispersed, they all converge on two areas. In particular, two mobility behaviors are noticeable: one while leaving the city and heading toward the airport, and another when leaving the city and heading for a location away from the city center. One can note that the patterns approaching the airport from North are higher than those from South. Another mobility pattern shown in Figure 6A is represented by a flow of people going from South-Center/sub-urban areas to the $\text{D}{\text{R}}_{19:00}^{3}$ region (i.e., parking lot). In fact, we can clearly recognize a route originating from the train station in the $\text{D}{\text{R}}_{12:00}^{12}$ region, or the ones starting from the South-Center. This flow could refer to people living outside the city that parked the cars in the parking lot to go to the city center and then coming back home in the suburbs after work. A second pattern, starting from the city center, going through the airport and ending to a sub-urban east area of Beijing, is shown in Figure 6B. In particular, there is a first mobility pattern from the airport to a suburban area, probably traced by people arriving to the airport and going back home in the residential sub-urban area. A second pattern represents the movement of people from the city center to the airport, e.g., going to work outside the city. Finally, Figure 6C shows a pattern from the airport to a train station in the city center. In particular, it is composed of three trips. The first one goes from the city center to the train station, the second one from a sub-urban area to the city center and the last one from the airport to a popular venue in the sub-urban South area of the city. More detailed results can be found in Cesario et al. (2017).

## 5. COVID-19 epidemic forecasting based on mobility patterns

An epidemic predictive approach based on spatial analysis, mobility and regressive models has been presented in Canino et al. (2022a). From movement and infection data, the approach is utilized to identify spatio- temporal predicted epidemic trends. The methodology's motivating premise is that infectious diseases propagate via human-to-human transmissions, making the study of spatio-temporal mobility data crucial for epidemic forecasting. Moreover, during an epidemic, the availability of accurate predictions can allow decision-makers in public health to forecast the spread of new cases and allow efficient resource planning for hospital needs and capacities. To assess the effectiveness of the approach in a real-world scenario, the experimental evaluation has been performed on mobility and COVID-19 data collected in the city of Chicago.

### 5.1. Approach

Let *T* = < *t*_1_, *t*_2_, …, *t*_*H*_> be an ordered list of timestamps. Let *ID* = {*id*_1_, *id*_2_, …, *id*_*M*_} be an *infection dataset*, where each infection record *id*_*h*_ = < (*x*_*h*_, *y*_*h*_, *t*_*h*_, *n*_*h*_) > is a tuple reporting the xy-localization (i.e., health center, hospital, etc.), timestamp and number of infection cases (i.e., number of positive cases). A *mobility dataset* *MD* = {τ_1_, τ_2_, ..., τ_*H*_} is a set of mobility traces (trajectories), where each trajectory τ_*h*_ = < (*x*_1*h*_, *y*_1*h*_, *t*_1_), …, (*x*_*nh*_, *y*_*nh*_, *t*_*h*_) > is a list of triples reporting the xy-position and timestamp. The goal of the approach is threefold. First, discover a set of *epidemic hotspots*, where an *epidemic hotspot* is a spatial area affected by higher density of infections than other areas, and involved in frequent mobility patterns. Second, discover a set of *epidemic patterns*, where each pattern is a couple < *EH*_*s*_, *EH*_*d*_ > (where *EH*_*s*_ and *EH*_*d*_ are two epidemic hotspots), meaning that the infection trend of *EH*_*s*_ influences the infection trend of *EH*_*d*_. Third, extract a function *F*_*spreading*_ that can predict the number of epidemic events (i.e., number of positive cases) in each *epidemic hotspot*.

**Figure 7:**
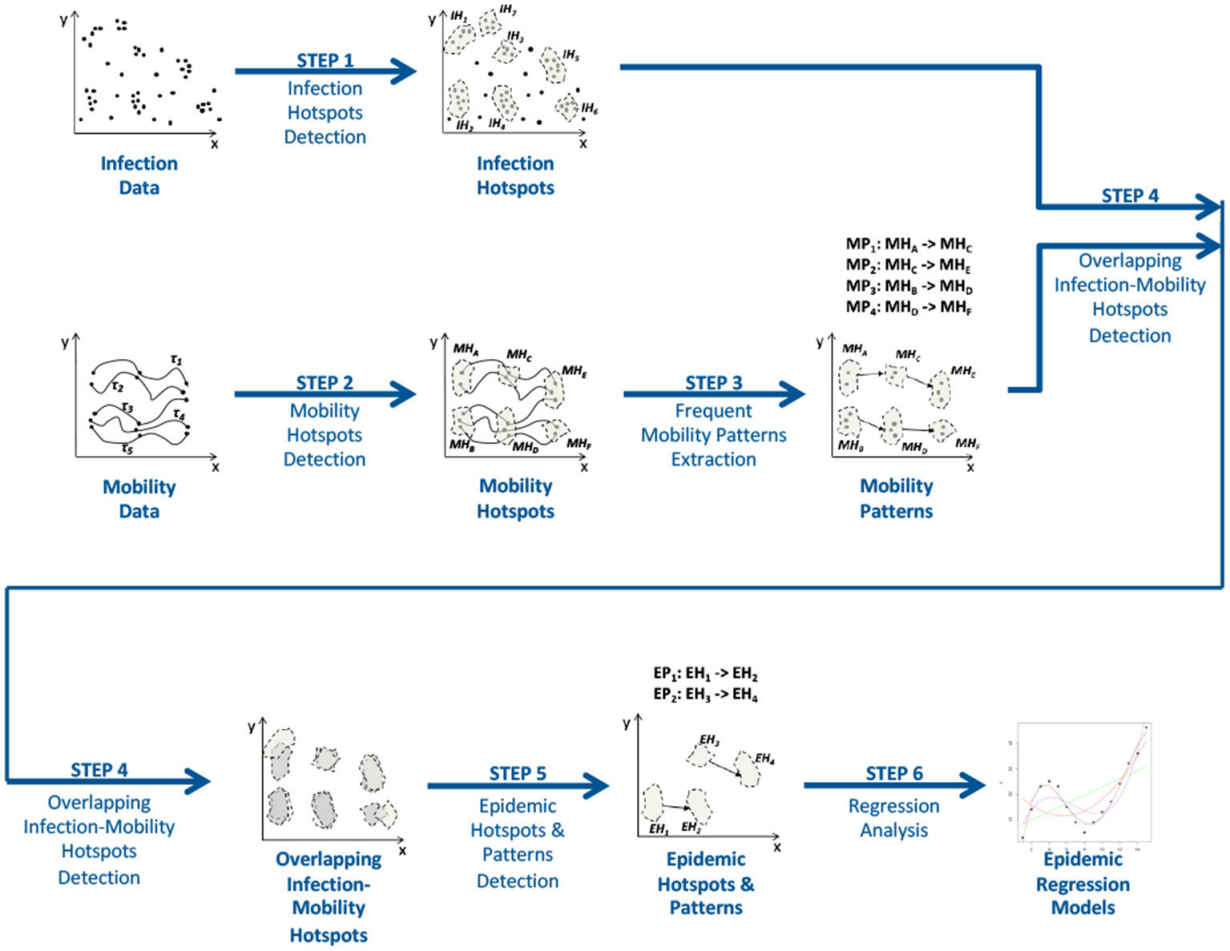
The approach workflow.

Figure 7 shows the workflow of the method, which has been designed to find predictive epidemic models from infections and mobility data. An array of epidemic hotspots, epidemic patterns, and epidemic regression models are produced by the algorithm after receiving infection and mobility data as input. The six steps that make up the workflow are listed below.

**Step 1 and 2: Detection of infection and mobility hotspots**. The execution of these two steps is aimed at discovering *Infection* and *Mobility Hotspots* from the two input datasets, Infection and Mobility datasets, respectively. In particular, a Mobility Hotspot is an urban area where mobility routes are more frequently traced than in other regions, whereas an Infection Hotspot is an urban area where infection events occur more frequently than in other places. The method uses the well-known density-based clustering algorithm DBScan, which is able to find these hotspots whose number and shapes are automatically detected by the algorithm.

**Step 3: Extraction of frequent mobility patterns**. This step has the goal to detect frequent patterns from mobility traces among the hotspots discovered at the previous step. Each mobility pattern is made up in a rule form, where the source mobility hotspot is the antecedent and the destination hotspot is the consequent of the rule. In particular, this task is performed by executing the T-Apriori (Cesario et al., 2017) algorithm.

**Step 4: Epidemic Hotspots Detection**. *Epidemic hotspots* are detected during this step. Specifically, an epidemic hotspot is defined as “an infection hotspots whose spatial overlap with a mobility hotspot is greater than a given threshold” (Canino et al., 2022a). The spatial overlap is calculated as the percentage of the overlapping area between the identified infection and mobility hotspots. Thus, an urban area that is both involved in a mobility pattern and characterized by an high density of infection cases is referred to as an epidemic hotspot.

**Step 5: Detection of epidemc patterns**. Given the epidemic hotspots detected during the previous step, epidemic patterns are extracted from the previously detected mobility patterns. When the source and destination of a mobility pattern are epidemic hotspots, the pattern is said to be epidemic.

**Step 6: Epidemic Spread Forecasting**. This step is aimed at extracting a specific epidemic forecasting model for each epidemic hotspot. In particular, for each destination epidemic hotspot in an epidemic pattern, a prediction model is trained by taking in consideration the infection data of such epidemic hotspot and its sources. This step can be implemented by exploiting LSTM artificial neural networks.

### 5.2. Experimental results

As test case study, the approach has been exploited to predict epidemic patterns in some Chicago neighborhoods. The goal of such tests comprises detecting the most significant mobility patterns among hotspots, the epidemic hotspots and epidemic predictive models. In particular, the final aim is to exploit the detected epidemic predictive models to estimate the number of epidemic events that are expected to occur in the future.

**Figure 8:**
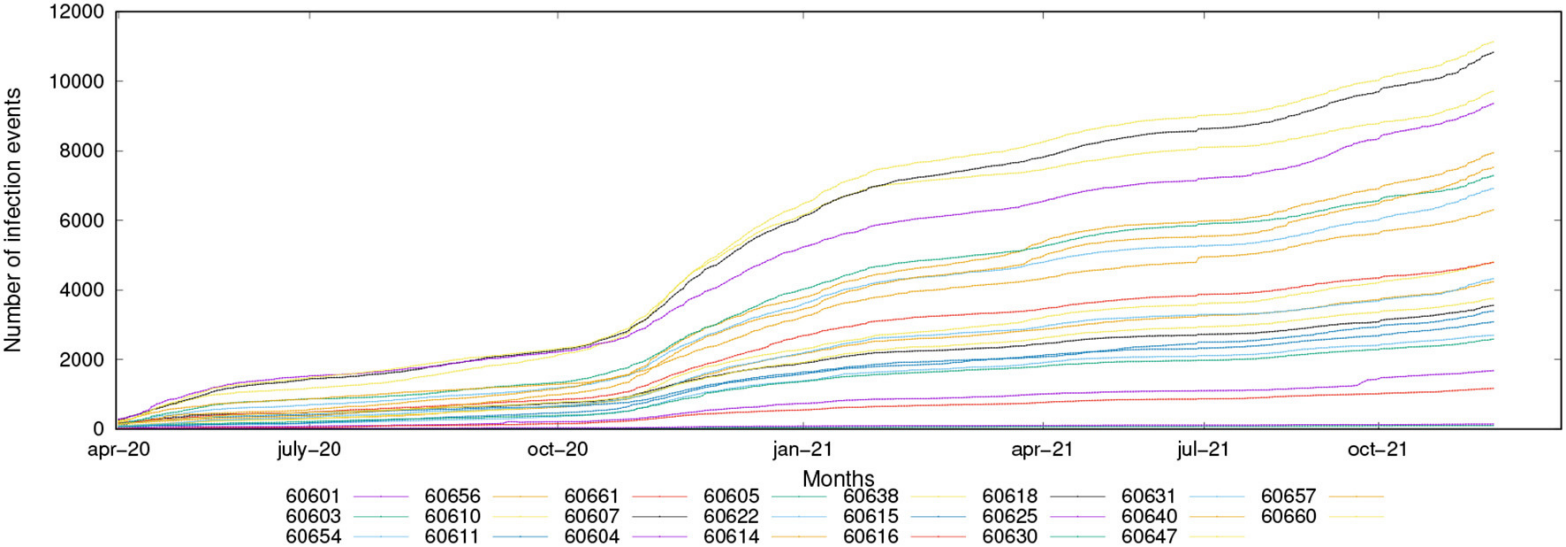
Cumulative number of infection cases vs. time for the evaluated Chicago ZIP codes.

The data used to extract the knowledge models and perform the experimental evaluation have been collected from real-world data repositories, covering the period from April 2020 to December 2021. Mobility data are composed of trajectories traced by vehicles, buses, pedestrians, while infection data are gathered from official daily COVID-19 data (cumulative number of positive cases, cumulative number of tested, etc.) (Canino et al., 2022a). Mobility data have been analyzed to discover mobility patterns and epidemic hotspots, while the infection data have been processed to discover predictive models for epidemic spread forecasting. Figure 8 shows the collected infection data (cumulative number of positive tested cases), grouped by zip-code. According to the plot, the incidence of infections is nearly stable in the Spring and Summer of 2020, climbs significantly in the late Autumn and Winter of 2020–2021, then stabilizes again in the Spring and Summer of 2021 before rising again in the Autumn 2021.

**Figure 9:**
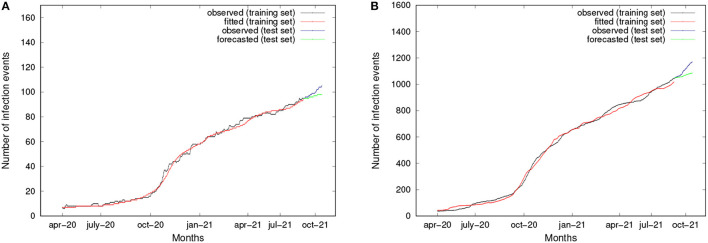
Cumulative number of infection events observed, fitted, and forecasted, for two zip codes. **(A)** ZipCode 60603. **(B)** ZipCode 60661.

Mobility patterns have been discovered by applying a pattern mining implementation of the well-known apriori algorithm (Cesario et al., 2017). Using infection data from the source locations as regression variables, the approach creates a specific epidemic forecasting model for each destination location after the detection of epidemic movement patterns. Forecasting models have been discovered by applying the LSTM algorithm, i.e., Long Short-Term Memory (Schmidhuber and Hochreiter, 1997), which is an artificial recurrent neural network used in deep learning and can process entire sequences of data. On the basis of actual data, the approach's experimental effectiveness has been assessed by computing how well the algorithm can forecast the daily occurrence of positive cases. The curves for two zip codes, 60603 and 60661, are shown in Figure 9. The observed and fitted data are plot in black and red, respectively. The training set period runs from April 2020 to August 2021. Observed and predicted data for the test set period, which ranges from September 2021 to December 2021, are represented by the colors blue and red, respectively. By examining the test set, we can see that the trends predicted by the LSTM models closely match those shown in the actual data. Finally, forecasting accuracy has been measured by several error indices. In particular, for all zip codes, the MAPE results lower than 10%, which appears to be a very interesting result. More details about the approach and achieved results can be found in Canino et al. (2022a,b).

## 6. Conclusion

This paper introduced how urban Big Data analysis can be exploited to design and develop data-driven smart city services. Then, it presented three real-case studies, showing how the application of data analysis to data-rich cities can provide innovative solutions to deal with urban issues. The first approach is aimed at detecting crime forecasting models, based on spatial analysis and auto-regressive models, which has been tested on crime events occurred in Chicago. The second one is methodology to discover mobility hotsposts and trajectory patterns from GPS data (tested on Beijing taxi traces). The third one is an approach to discover spatio-temporal predictive epidemic patterns from mobility and infection data, whose experimental evaluation has been carried out on real-world COVID-19 data. The presented real-world cases prove that data analytics models can effectively support city managers in tackling smart city challenges and improving urban applications.

As further research challenges smart cities have to deal with in the future, there are several opportunities that are promising and relevant in the smart city domain, including the following ones:

- *Improving efficiency and effectiveness of city network communications*. The transformation from an urban metropolitan area toward a smart city is strictly dependent on its communication network, which must be more pervasive and efficient to make all monitoring and analysis devices (sensors, computing nodes, smart objects) working together in a collaborative digital ecosystem.
- *More pervasive use of data*. With a more connected city, data can be more freely created and shared, to improve services and introduce more innovation. The increasing pervasiveness of data can be exploited by modern machine and deep learning algorithms to proficiently solve urban issue.
- *More workable policies with legislation*. An important challenge to be addressed is finding workable policies to regulate interactions among city managers, urban data scientists and ICT stakeholders to collaborate in research-and-development investments, aimed at implementing innovative services for citizens.

## Data availability statement

The raw data supporting the conclusions of this article will be made available by the authors, without undue reservation.

## Author contributions

EC: study conception and design, data collection, analysis and interpretation of results, and manuscript preparation.
